# Comparative Microbiota and Metabolite Profiles of Undried and Dried Typica Luwak (Civet) Coffee Beans

**DOI:** 10.3390/foods15081334

**Published:** 2026-04-11

**Authors:** Mengjie Li, Xianwen Wang, Liyan Ma, Kunlun Huang, Tao Tong

**Affiliations:** 1 College of Food Science and Nutritional Engineering, China Agricultural University, Beijing 100083, China; mengjieli1112@163.com (M.L.); lyma1203@cau.edu.cn (L.M.); foodsafety66@cau.edu.cn (K.H.); 2 Baoshan Inspection and Testing Institute, Baoshan 678000, China; awenchina@163.com; 3 Key Laboratory of Coffee Quality and Safety, State Administration for Market Regulation, Baoshan 678000, China; 4 Key Laboratory of Safety Assessment of Genetically Modified Organism (Food Safety), The Ministry of Agriculture and Rural Affairs of the People’s Republic of China, Beijing 100083, China; 5 Beijing Laboratory for Food Quality and Safety, Beijing 100083, China

**Keywords:** Kopi Luwak (civet coffee), typica coffee, dry processing, microbiome, metabolome

## Abstract

Kopi Luwak (civet coffee), produced from coffee beans recovered from the feces of the Asian palm civet, is among the most expensive specialty coffees worldwide for its unique sensory characteristics. In this study, a multi-omics strategy was employed to elucidate the impact of drying on the microbial community structure and metabolic profiles of Typica Kopi Luwak beans. Drying induced pronounced shifts in the microbial composition, with a significant enrichment of *Sphingobacterium* and depletion of *Streptococcus* at the genus level. Concurrently, drying resulted in substantial metabolic remodeling, characterized by increased levels of prenol lipids, fatty acyls, carboxylic acids and derivatives, steroids and steroid derivatives, and organooxygen compounds, accompanied by a marked reduction in flavonoids. KEGG pathway analysis indicated that both altered microbial taxa and metabolites were associated with lipid metabolism, carbohydrate metabolism, amino acid metabolism, and the biosynthesis of other secondary metabolites. Correlation network analysis further revealed the associations between key microbial genera and specific classes of differential metabolites. Collectively, these findings suggest the potential role of post-excretion sun-drying in shaping the microbiome and metabolome of Typica Kopi Luwak beans, offering a scientific basis for controlled or in vitro fermentation strategies to produce coffees with reproducible quality attributes.

## 1. Introduction

Coffee is one of the world’s most economically important agricultural commodities, and its global popularity is linked to its desirable flavor profile [1]. The quality and final sensory attributes of coffee are determined not only by genetic factors and terroir but also significantly by postharvest processing methods [2]. Traditional processing techniques, such as wet (washed) and dry (natural) methods, fundamentally rely on controlled or spontaneous microbial fermentation to remove the mucilage and pulp [3]. This crucial step involves microbial communities transforming the fruit’s carbohydrates, proteins, and organic acids, thus producing essential flavor precursors that ultimately shape the characteristic aroma and taste of the roasted bean [2]. Understanding and optimizing this microbial contribution is vital for advancing the science of coffee quality control.

Kopi Luwak (civet coffee) is globally recognized for its exceptional rarity and controversial, yet distinct, flavor [4]. This unique coffee is processed internally within the digestive tract of the Asian palm civet. After the civet ingests mature coffee cherries, the beans undergo a profound endogenous fermentation catalyzed by the animal’s powerful digestive enzymes and, more importantly, its specific gut microbial community. This process reportedly results in a coffee with characteristics such as reduced bitterness, smoother mouthfeel, and unique notes of chocolate and earthiness [5,6]. In addition, the gastrointestinal bioprocessing undergone by Kopi Luwak results in distinct chemical compositions. Febrina et al. reported that, relative to conventional green Arabica coffee beans, green Kopi Luwak beans contained higher concentrations of alanine, citric acid, lactic acid, malic acid, and trigonelline [7]. Further comparative metabolomic analyses of roasted coffee beans revealed that roasted Kopi Luwak exhibited elevated levels of acetic acid, lipids, trigonelline, quinic acid, citric acid, malic acid, guaiacol derivatives, as well as pyrazine and furan compounds, compared with conventionally roasted coffee [5,8]. Moreover, a recent study demonstrated that the concentrations of mineral elements such as Cu and Rb, along with total phenolic content, were significantly higher in Kopi Luwak than in ordinary coffee [9].

In addition to the gastrointestinal digestion within the civet, post-excretion sun-drying represents another critical stage influencing the chemical composition of Kopi Luwak beans [10]. However, current research on drying processes has largely focused on conventional coffee [11,12], and the microbial and metabolic dynamics during the drying of civet coffee beans remain poorly understood. We hypothesize that the drying process not only preserves a fraction of gut-derived microbiota but also facilitates the colonization and enrichment of environmental microorganisms, thereby jointly driving metabolic transformations. Moreover, exposure to open environmental conditions during sun-drying may introduce external microbial contaminants, including taxa of potential hygienic concern. Therefore, elucidating microbial dynamics during this stage is important for understanding flavor development as well as from a food safety perspective.

Accordingly, the present study was designed to investigate the impact of post-excretion sun-drying on microbial community dynamics and metabolic remodeling in Kopi Luwak beans and to establish a comprehensive association between microbial shifts and corresponding metabolic alterations by using a multi-omics approach.

## 2. Materials and Methods

### 2.1. Sample Collection and Preparation

The Typica (*C. arabica*) Kopi Luwak (civet coffee) samples used in this study were provided by Maonika Coffee Co., Ltd. (Baoshan, China). Specifically, civet coffee beans were obtained from seven independent Asian palm civets maintained under standardized feeding conditions. Freshly excreted coffee bean samples were collected from each animal, and each biological replicate (*n* = 7) corresponded to an independent sample derived from a single civet. All samples were collected from the same production batch under uniform management conditions. After collection, the samples were homogenized within each replicate and divided into two portions for paired analysis: one portion was immediately processed and designated as Typica_fresh (undried Typica Kopi Luwak beans), while the other portion was subjected to sun-drying and designated as Typica_dry (dried Typica Kopi Luwak beans), ensuring direct comparability between undried and dried samples.

The sun-drying process was conducted under natural environmental conditions for approximately 2~3 weeks, with an average ambient temperature of 20 °C. Samples were exposed to direct sunlight during the daytime and turned several times a day to ensure uniform drying until the moisture content reached approximately 10% (*w*/*w*).

Prior to analysis, visible debris and residual fecal materials were carefully removed under sterile conditions. No washing step was performed to preserve the native microbial communities. To minimize contamination, the whole bean samples were handled using sterile tools and were immediately transferred into sterile containers and stored at −80 °C until further analysis.

### 2.2. Microbiome Sequencing and Bioinformatic Analysis of Bacterial Communities

#### 2.2.1. DNA Extraction and Polymerase Chain Reaction (PCR) Amplification

Genomic DNA was extracted from all Kopi Luwak samples following the manufacturer’s protocol. The resulting DNA quality and concentration were verified through standard methodologies: quality assessment via 1.0% agarose gel electrophoresis and quantification using a NanoDrop 2000 spectrophotometer (Thermo Scientific, Waltham, MA, USA). Purified DNA extracts were stored at −80 °C until downstream molecular analysis. The hypervariable V3–V4 region of the bacterial 16S rRNA gene was targeted for sequencing. Amplification was performed using the universal primer set 338F (5′-ACTCCTACGGGAGGCAGCAG-3′) and 806R (5′-GGACTACHVGGGTWTCTAAT-3′). The PCR was executed in a T100 Thermal Cycler (BIO-RAD, Hercules, CA, USA). Following amplification, the PCR products were purified using the PCR Clean-Up Kit (Yuhua, Shanghai, China) and subsequently quantified with a Qubit 4.0 Fluorometer (Thermo Scientific Waltham, MA, USA).

#### 2.2.2. Illumina Sequencing

The purified amplicons were pooled in equimolar concentrations and subjected to paired-end sequencing on an Illumina NextSeq 2000 platform (Illumina, San Diego, CA, USA). Sequencing was carried out according to the standard operating protocols by Majorbio Bio-Pharm Technology Co., Ltd. (Shanghai, China).

Following demultiplexing, the raw sequencing reads underwent stringent quality filtering using fastp (version 0.19.6) to remove low-quality data. The filtered paired-end reads were subsequently merged utilizing FLASH (version 1.2.11). High-throughput sequencing was then processed for error correction and de-noising using the DADA2 plugin within the QIIME2 pipeline (version 2024), employing recommended parameters. DADA2 leverages an error profile model to yield single-nucleotide resolution, generating Amplicon Sequence Variants (ASVs). The taxonomic assignment for all identified ASVs was performed using the Naive Bayes consensus taxonomy classifier implemented in QIIME2, against the latest SILVA 16S rRNA database (version 138.2). To minimize the influence of host- and plant-derived DNA on microbial community analysis, ASVs assigned to chloroplasts and mitochondria were explicitly identified and removed from the dataset prior to downstream analyses.

#### 2.2.3. Microbiota Data Analysis

Bioinformatic processing of the microbial data was conducted using the Majorbio Cloud platform (https://cloud.majorbio.com (accessed on 19 December 2025)). The α-diversity indices, including Ace, Shannon, Pielou’s evenness, and phylogenetic diversity (Pd) index, were calculated using Mothur (version 1.30.1) based on the normalized ASV table. To evaluate the structural similarity among microbial communities across different samples, Principal Component Analysis (PCA) was performed based on Euclidean distance metrics using the Vegan (version 3.3.1) R package. Furthermore, the Analysis of Similarities (ANOSIM) test was implemented within the same package to quantify the extent of variation attributed to the treatment and to determine its statistical significance.

Relative abundances of bacterial phyla and genera were visualized using stacked bar plots, with taxa representing < 2% of the total abundance grouped as “Others”. Differential phyla and genera between the two groups were preliminarily identified using the Wilcoxon rank-sum test with false discovery rate (FDR) correction.

To identify differentially abundant bacterial taxa (from phylum to genus levels) between the study groups, linear discriminant analysis (LDA) effect size (LEfSe) was employed. Significant biomarkers were selected based on a logarithmic LDA score threshold of >3.5 and *p* < 0.05. Given the previously reported high false-positive rate associated with LEfSe [13], we additionally applied Analysis of Composition of Microbiomes (ANCOMs) using the ANCOM 4.0.2 plugin in QIIME 2 to further refine the identification of bacterial taxa enriched between groups. The PICRUSt was used to predict the functional profiles of microbial communities at level 2. Statistically significant differences were evaluated by STAMP (http://kiwi.cs.dal.ca/Software/STAMP (accessed on 29 December 2025)).

### 2.3. Untargeted Metabolomics

#### 2.3.1. Sample Preparation

Untargeted metabolomic profiling of Kopi Luwak samples was conducted by Shanghai Majorbio Bio-pharm Technology Co., Ltd. Metabolites were extracted using 400 μL of methanol (4:1, *v*/*v*) containing 0.02 mg/mL L-2-chlorophenylalanine as an internal standard. The extracts were incubated at −10 °C, homogenized using a Wonbio High-Throughput Tissue Crusher at 50 Hz for 6 min, and subsequently sonicated at 40 kHz for 30 min at 5 °C. Protein precipitation was achieved by incubation at −20 °C for 30 min, followed by centrifugation at 13,000× *g* for 15 min at 4 °C. The resulting supernatants were collected and transferred to autosampler vials for liquid chromatography–tandem mass spectrometry (LC–MS/MS) analysis.

#### 2.3.2. LC–MS/MS Analysis

LC–MS/MS measurements were performed using a Thermo UHPLC-Q Exactive HF-X system fitted with an ACQUITY HSS T3 column (100 mm × 2.1 mm, 1.8 μm; Waters, Milford, MA, USA). Mass spectra were acquired on a Q Exactive HF-X mass spectrometer equipped with an electrospray ionization source operating in both positive and negative ion modes. Raw LC–MS data were processed and converted into a standardized format using Progenesis QI software (version 2.3, Waters, Milford, CT, USA).

#### 2.3.3. Data Processing and Metabolite Annotation

Metabolite annotation in this study was primarily based on accurate mass matching and MS/MS spectral comparison, which corresponds to Level 2 identification according to the Metabolomics Standards Initiative. The Human Metabolome Database (HMDB; https://hmdb.ca (accessed on 22 July 2025)), the most comprehensive organism-specific metabolite database, was used for metabolite classification. Metabolites were categorized according to HMDB hierarchical levels (class or subclass), and the names and corresponding metabolite counts were presented in descending order. The resulting data matrix was uploaded to the Majorbio cloud platform (https://cloud.majorbio.com (accessed on 22 July 2025)) for downstream analysis.

#### 2.3.4. Statistical and Pathway Analysis

Following normalization to total ion intensity, the dataset was subjected to multivariate statistical analysis using the Ropls R package (version 1.6.2). Variations in metabolite composition and abundance among samples were quantified based on inter-sample correlation analyses, and a correlation heatmap was generated to visualize metabolic similarity. Partial least squares discriminant analysis (PLS-DA) was employed to assess metabolic differences among experimental groups. Venn diagrams and UpSet plots were used to summarize the numbers of shared and unique metabolites across groups.

Differential metabolites were annotated based on a variable importance in projection (VIP) score > 1.0, *p* < 0.05, and a fold change (|FC|) ≥ 2. These metabolites were subjected to pathway enrichment analysis using the Kyoto Encyclopedia of Genes and Genomes (KEGG) database to identify significantly affected metabolic pathways. *p*-values and pathway impact values were calculated to reflect the relative importance of metabolites within each pathway, integrating both pathway topology and metabolite abundance changes.

The HMDB class level was used to classify the differential metabolites; the relative abundances of the six major classes were visualized using a heatmap, while information on the remaining representative metabolites is provided in Table S2.

### 2.4. Multi-Omics Integration Network Analysis

Integrated multi-omics analysis was performed to investigate associations between the microbiome and metabolome. Genus-level microbial taxa and significantly altered metabolites were selected for correlation analysis. Spearman’s rank correlation coefficients were calculated using the OmicStudio (https://www.omicstudio.cn (accessed on 31 December 2025)) [14], with statistical significance defined as *p* < 0.05. Correlation coefficients (r) ≥ 0.7 were considered indicative of strong positive co-abundance relationships, whereas r ≤ −0.7 were interpreted as strong negative associations. Statistically significant correlations were imported into Cytoscape (version 3.10.4) for construction and visualization of the interaction network.

### 2.5. Statistical Analysis

The statistical analyses and graphical representations were performed using GraphPad Prism (version 9.0). Data are presented as mean ± standard error of the mean (SEM). Comparisons between two groups were conducted using an unpaired two-tailed Student’s *t*-test, while multiple comparisons were performed using the Wilcoxon rank-sum test with FDR correction. Statistical significance was defined as *p* < 0.05.

## 3. Results

### 3.1. Difference in Microbiota Profiles Between Undried and Dried Kopi Luwak Beans

#### 3.1.1. Microbial Community Diversity

**Figure 1:**
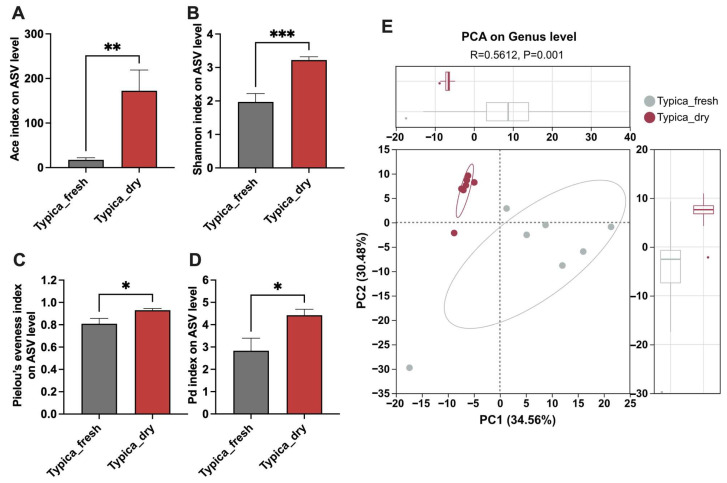
Effects of drying on the microbial diversity of Typica Kopi Luwak beans. (**A**) Ace index. (**B**) Shannon index. (**C**) Pielou’s evenness index. (**D**) Pd index. (**E**) PCA of the microbiota based on Euclidean distance metrics (ANOSIM, R = 0.5612, *p* = 0.001) of microbiota. *n* = 7. * *p* < 0.05, ** *p* < 0.01, *** *p* < 0.001.

The α-diversity reflects species richness and evenness within a microbial community. To analyze the effect of the drying process on the α-diversity of microbiota in Typica civet coffee beans, several alpha diversity indices were used, including Ace (richness estimator), Shannon (diversity index accounting for both richness and evenness), Pielou’s evenness (community evenness), and Pd, which reflects the phylogenetic breadth of the microbial community. The results showed that, compared with undried Typica Kopi Luwak beans, dried Kopi Luwak exhibited significantly higher Ace, Shannon, Pielou’s evenness, and Pd index, suggesting a higher α-diversity in dried samples (Figure 1A–D).

β-diversity describes compositional differences among microbial communities across ecological samples. In the present study, PCA based on Euclidean distance metrics was used to visualize inter-group variation. As shown in Figure 1E, the first two principal coordinates (PC1 and PC2) account for the major differences among the samples. A clear separation was observed between samples collected before and after the drying process, and this distinction was confirmed to be statistically significant by ANOSIM (*p* < 0.05).

#### 3.1.2. Microbial Community Structures

**Figure 2:**
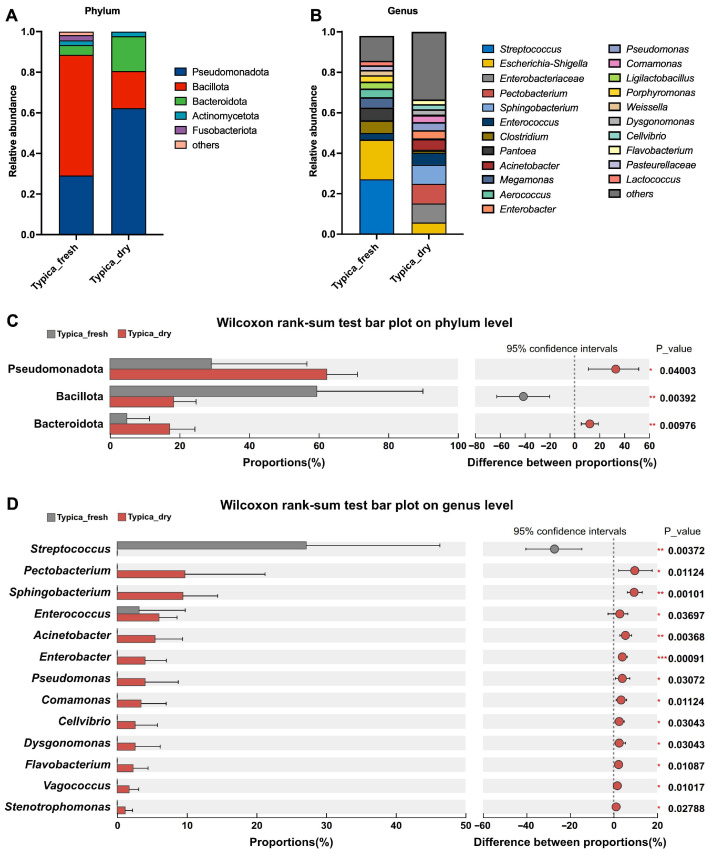
Effects of drying on the microbial community structures of Typica Kopi Luwak beans. Taxonomic proportions at the (**A**) phylum and (**B**) genus levels. Differential microbial taxa at the (**C**) phylum and (**D**) genus levels between Typica_fresh and Typica_dry groups were identified using the Wilcoxon rank-sum test with FDR correction. *n* = 7. * *p* < 0.05, ** *p* < 0.01, *** *p* < 0.001.

We compared the microbial community structures of Typica Kopi Luwak samples before and after drying at both the phylum and genus levels (Figure 2). The results revealed that the undried and dried samples exhibited marked differences in microbial composition. At the phylum level, Pseudomonadota and Bacillota were the dominant phyla in both groups, followed by Bacteroidota, Actinomycetota, and Fusobacteriota (Figure 2A). At the genus level, the microbial community of undried Kopi Luwak beans was dominated by *Streptococcus*, with relatively abundant genera also including *Clostridium*, *Pantoea*, and *Megamonas*. In contrast, the dried beans exhibited a more evenly distributed microbial profile across multiple genera, including *Enterobacteriaceae*, *Pectobacterium*, *Sphingobacterium*, and *Enterococcus*, with a higher proportion of genera accounting for <2% of total abundance, reflecting an increase in microbial diversity after drying (Figure 2B).

To identify differential taxa associated with the drying process, two-group comparisons were performed with multiple testing correction. At the phylum level, drying significantly increased the relative abundances of Pseudomonadota and Bacteroidota, while significantly decreasing Bacillota (Figure 2C). At the genus level, only *Streptococcus* was significantly reduced in the dried beans, whereas the relative abundances of *Pectobacterium*, *Sphingobacterium*, *Enterococcus*, *Acinetobacter*, *Enterobacter*, *Pseudomonas*, *Comamonas*, *Cellvibrio*, *Dysgonomonas*, and *Flavobacterium* were significantly increased (Figure 2D).

#### 3.1.3. Microbial Key Phylotypes

**Figure 3:**
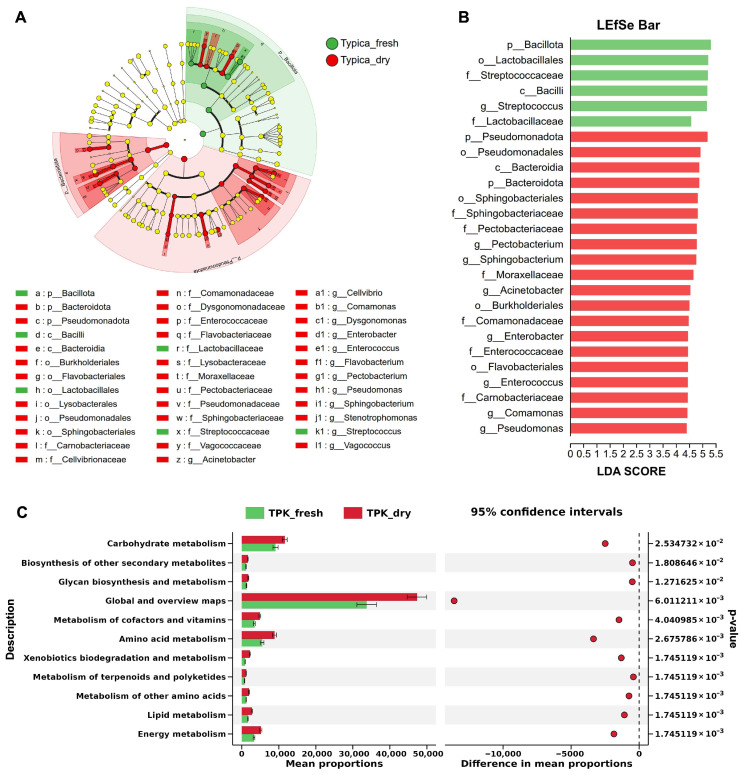
Identification and functional characterization of key differential microorganisms in Kopi Luwak beans before and after drying. (**A**) LEfSe-generated cladograms illustrating differences in bacterial taxa between the Typica_fresh and Typica_dry groups. (**B**) LDA scores showing bacterial taxa with significantly different abundances between the Typica_fresh and Typica_dry groups (LDA > 3.5). (**C**) Predicted functional metabolic profiles of the microbial communities based on KEGG metabolic pathways in the Typica_fresh and Typica_dry groups. Statistical significance was assessed using the Wilcoxon rank-sum test in STAMP (*p* < 0.05). *n* = 7.

Next, LDA integrated with LEfSe was applied to identify ASVs that most strongly contributed to the observed differences in microbial community composition. Under the thresholds of LDA > 3.5 and *p* < 0.05, a total of three phyla, two classes, six orders, fourteen families, and thirteen genera were identified as differentially abundant between the undried and dried Kopi Luwak bean samples (Figure 3A,B). Notably, taxa enriched in the Typica_fresh group were exclusively affiliated with the phylum Bacillota, including *Streptococcus* at the genus level. In contrast, taxa enriched in the Typica_dry group were mainly assigned to Pseudomonadota (including *Acinetobacter*, *Pseudomonas*, *Cellvibrio*, *Comamonas*, *Enterobacter*, and *Pectobacterium*) and Bacteroidota (including *Flavobacterium*, *Sphingobacterium*, and *Dysgonomonas*). In addition, LEfSe identified two genera (*Enterococcus* and *Vagococcus*) that were significantly increased in the Typica_dry group, both belonging to the phylum Bacillota (Figure 3A,B). Hence, the LEfSe results corroborated the findings from the two-group comparison analysis and provided a systematic overview of the taxonomic relationships among the differential microbial communities.

ANCOM is a statistical method for detecting significant differences between groups that does not rely on data distribution assumptions. This feature allows ANCOM to overcome some of the limitations of the LEfSe method, particularly in reducing the risk of false positives in relative abundance analysis, thereby providing more robust statistical results [13]. To further investigate microbial community shifts in Typica Kopi Luwak beans before and after drying, we applied the ANCOM analysis available in QIIME2. The results revealed that *Streptococcus* and *Sphingobacterium* were identified as differentially abundant genera between the two groups, with their abundance significantly downregulated and upregulated in the dried Typica Kopi Luwak beans, respectively (Table 1). Collectively, these findings suggest that the drying process significantly alters the microbiome composition of Typica Kopi Luwak beans.

#### 3.1.4. Predicted Metabolic Profiles of the Microbiota

The KEGG functional profiles of the microbiota were predicted using PICRUSt2, based on 16S rRNA gene amplicon sequencing data. Several metabolic pathways showed significant alterations in response to the drying process in Typica Kopi Luwak beans. As shown in Figure 3C, pathways involved in carbohydrate metabolism, biosynthesis of other secondary metabolites, glycan biosynthesis and metabolism, metabolism of cofactors and vitamins, amino acid metabolism, xenobiotics biodegradation and metabolism, metabolism of terpenoids and polyketides, metabolism of other amino acids, lipid metabolism, and energy metabolism were all significantly enriched in the microbiota of dried Typica Kopi Luwak beans. These results suggest that the drying process may alter the metabolic functional potential of the microbial community.

### 3.2. Difference in Metabolite Profiles Between Undried and Dried Kopi Luwak Beans

#### 3.2.1. Metabolite Profiles Analysis

**Figure 4:**
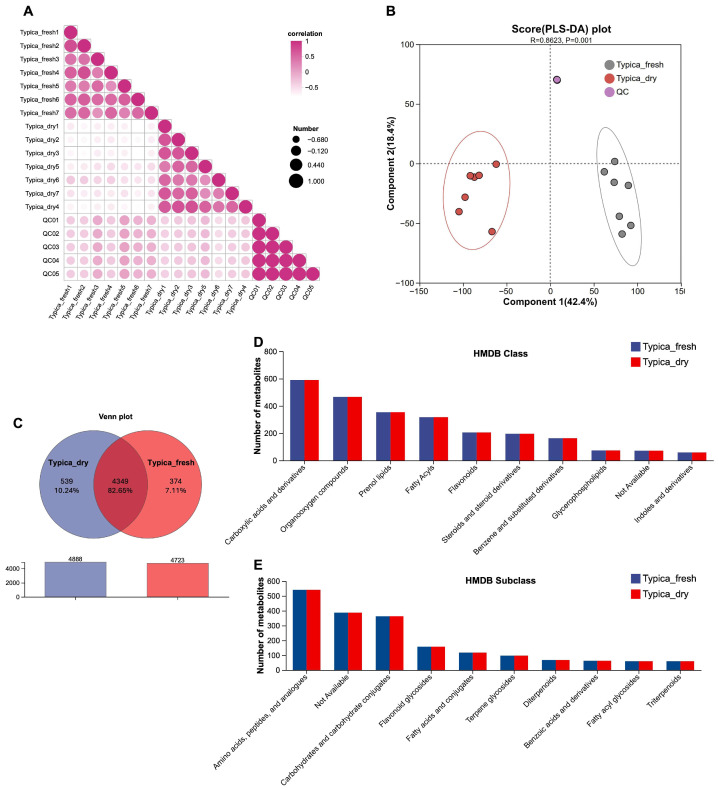
Effects of drying on the metabolic profiles of Typica Kopi Luwak beans. (**A**) Heatmap showing correlations among samples based on metabolite profiles. (**B**) PLS-DA illustrating metabolic differences between the Typica_fresh and Typica_dry groups (ANOSIM, R = 0.8623, *p* = 0.001). The R value refers to the ANOSIM (Analysis of Similarities) statistic calculated based on the metabolomics distance matrix, with values closer to 1 indicating greater between-group dissimilarity. (**C**) Venn diagram depicting the number of metabolites identified in the Typica_fresh and Typica_dry groups and their overlap. Distribution of identified metabolites from the Typica_fresh and Typica_dry groups at the HMDB class (**D**) and subclass (**E**) levels. *n* = 7.

To investigate changes in the metabolic profiles of Typica Kopi Luwak beans during the drying process, untargeted metabolomic analysis was performed. Inter-sample correlation analysis was used to quantify variations in metabolite composition and abundance among samples, with correlation coefficients approaching 1 indicating higher metabolic similarity. As shown in Figure 4A, strong correlations were observed among samples within each group, whereas correlations between the Typica_fresh and Typica_dry groups were markedly lower. These results indicate substantial alterations in metabolite composition and abundance following the drying process.

Consistently, the PLS-DA score plot showed a clear separation between the two groups (R = 0.8623, *p* = 0.001) (Figure 4B), and model validation parameters (Table S1), together with permutation testing (Figure S1), suggested acceptable robustness and stability of the model. Venn diagram analysis was further used to quantify shared and unique metabolites between groups. A total of 4888 and 4723 metabolites were annotated in the Typica_fresh and Typica_dry groups, respectively, with 4349 metabolites (82.65%) shared between the two groups (Figure 4C). In addition, 374 metabolites (7.11%) were unique to the Typica_fresh group, whereas 539 metabolites (10.24%) were specific to the Typica_dry group (Figure 4C).

The Human Metabolome Database (HMDB), a widely recognized reference resource for metabolite identification and annotation, was subsequently used to classify the annotated features. At the HMDB class level, the predominant categories included carboxylic acids and derivatives, organooxygen compounds, prenol lipids, fatty acyls, flavonoids, steroids and steroid derivatives, benzene and substituted derivatives, glycerophospholipids, and indoles and derivatives (Figure 4D). Further subclass-level annotation classified metabolites primarily into amino acids, peptides, and analogs; carbohydrates and carbohydrate conjugates; flavonoid glycosides; fatty acids and conjugates; terpene glycosides; diterpenoids; benzoic acids and derivatives; fatty acyl glycosides; and triterpenoids (Figure 4E).

#### 3.2.2. Differential Metabolite Analysis

**Figure 5:**
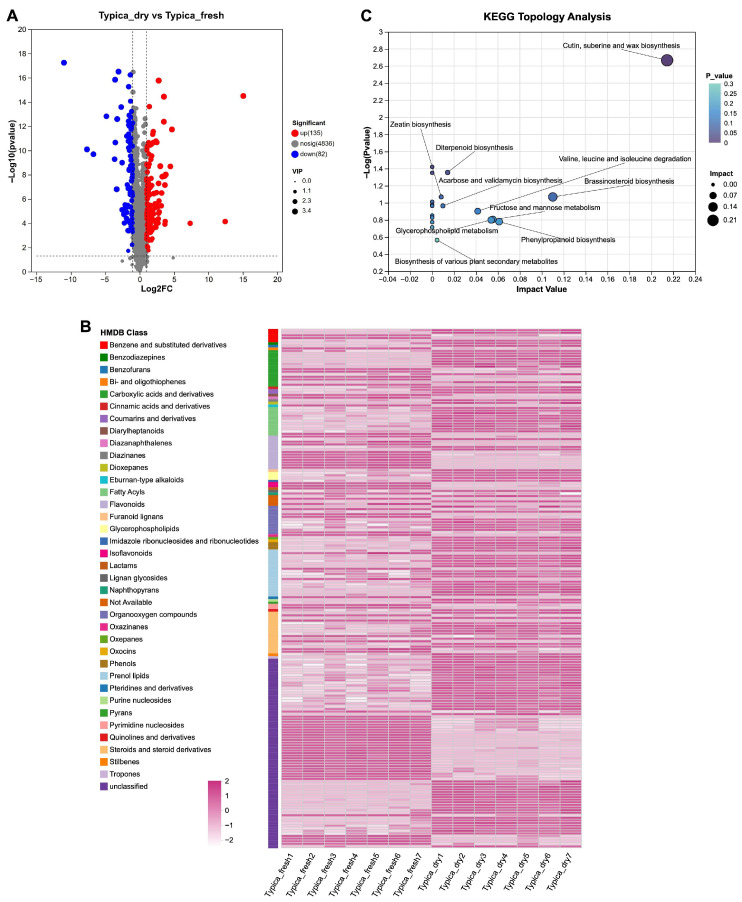
Annotation and pathway enrichment analysis of differential metabolites in Kopi Luwak beans before and after drying. (**A**) Volcano plots showing differential metabolites between the Typica_fresh and Typica_dry groups. (**B**) Heatmap illustrating the relative abundances of differential metabolites between the two groups at the HMDB class level. (**C**) Metabolic pathway topology analysis of differential metabolites between the two groups. Each bubble represents a KEGG pathway. The x-axis indicates the pathway impact value, reflecting the relative importance of metabolites within the pathway. The y-axis represents the enrichment significance of metabolites in each pathway, expressed as −log(*p*-value). Bubble size corresponds to the impact value, with larger bubbles indicating greater pathway importance. *n* = 7.

To characterize metabolic alterations induced by the drying process, differential metabolite analysis was conducted. Based on the criteria of VIP > 1 derived from the PLS-DA model, *p* < 0.05, and |FC| ≥ 2, a total of 217 differential metabolite features were found between the Typica_fresh and Typica_dry groups (Figure 5A). Specifically, compared with undried Typica Kopi Luwak beans, 135 metabolites were significantly enriched, whereas 82 metabolites were significantly depleted following the drying process (Figure 5A).

These 217 differential metabolites were subsequently annotated and classified using the HMDB. At the class level, 138 metabolites were successfully annotated and were primarily assigned to compound categories including benzodiazepines, fatty acyls, flavonoids, organooxygen compounds, and steroids and steroid derivatives (Figure 5B). A heatmap constructed based on the relative abundances of these 138 annotated metabolites revealed distinct metabolic patterns between the Typica_fresh and Typica_dry groups, further highlighting the pronounced impact of drying on the metabolic profiles of Typica Kopi Luwak beans (Figure 5B).

#### 3.2.3. Pathway Enrichment Analysis of Metabolic Pathways

To further elucidate the biological pathways involved in the metabolism of differential metabolites and their potential functional implications, KEGG pathway topology analysis was performed. Based on the 217 differential metabolite features between the Typica_fresh and Typica_dry groups, a total of 20 significantly altered metabolic pathways were detected (Figure 5C). Among these, the top 10 pathways ranked by impact value included cutin, suberine and wax biosynthesis; brassinosteroid biosynthesis; phenylpropanoid biosynthesis; glycerophospholipid metabolism; fructose and mannose metabolism; valine, leucine and isoleucine degradation; diterpenoid biosynthesis; acarbose and validamycin biosynthesis; zeatin biosynthesis; and the biosynthesis of various plant secondary metabolites (Figure 5C).

#### 3.2.4. Classification of Differential Metabolites

**Figure 6:**
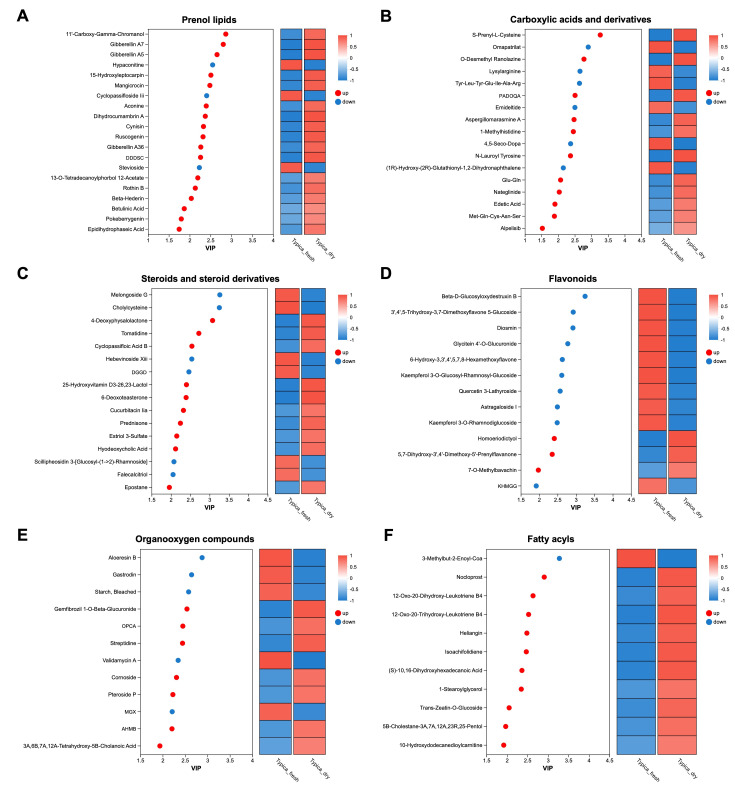
Distribution of VIP scores for differential metabolites across major HMDB classes. The six classes with the highest number of identified metabolites are shown, including (**A**) prenol lipids, (**B**) carboxylic acids and derivatives, (**C**) steroids and steroid derivatives, (**D**) flavonoids, (**E**) organooxygen compounds, and (**F**) fatty acyls. VIP scores were derived from the PLS-DA model and reflect the relative contribution of each metabolite to group discrimination. DDDSC, (12S,15S)-15-O-Demethyl-10,29-Dideoxy-11,12-Dihydro-Striatin C. PADOQA, Pentanoic Acid, 5-(Dipentylamino)-5-Oxo-4-((3-Quinolinylcarbonyl)Amino)-, (R)-. DGGD, Digitoxigenin 3-[Glucosyl-(1->6)-Glucosyl-(1->4)-2,6-Dideoxyribohexoside]. KHMGG, Kaempferol 3-[6′′-(3-Hydroxy-3-Methylglutaryl)Glucoside]-7-Glucoside. OPCA, (Z)-3-Oxo-2-(2-Pentenyl)-1-Cyclopenteneacetic Acid. MGX, 2-O-(4-O-Methyl-A-D-Glucopyranuronosyl)-D-Xylose. AHMB, 1-(5-Acetyl-2-Hydroxyphenyl)-3-Methyl-1-Butanone.

Comparative analysis of the metabolic profiles between Typica_fresh and Typica_dry revealed the variation trends of differential metabolites across specific HMDB class categories. Figure 6 presents the VIP scores and abundance heatmap for metabolites in six of the most abundant categories (89 differential metabolites) between the two groups. Notably, the content of nearly all prenol lipids (Figure 6A) and fatty acyls (Figure 6F), as well as the majority of carboxylic acids and derivatives (Figure 6B), steroids and steroid derivatives (Figure 6C), and organooxygen compounds (Figure 6E), was significantly increased following the drying process. In contrast, most flavonoid compounds exhibited a significant decrease in abundance after drying (Figure 6D).

In addition, several other metabolites exhibited changes in relative abundance between the undried and dried samples. Owing to the relatively small number of metabolites within these classes, they are summarized in Table S2. For example, phenols (e.g., adipostatin A, 2-methoxy-4-vinylphenol, coniferyl alcohol), coumarins and derivatives (e.g., 6′-O-formylmarmin, epoxybergamottin), and cinnamic acids and derivatives (e.g., 16-feruloyloxypalmitate) were significantly enriched in the dried samples, while isoflavonoids (e.g., biochanin A 7-(6-malonylglucoside), 6′-malonyltrifolirhizin) showed significant depletion after drying.

### 3.3. Correlation Between Microbiota and Metabolites Regulated by Drying

**Figure 7:**
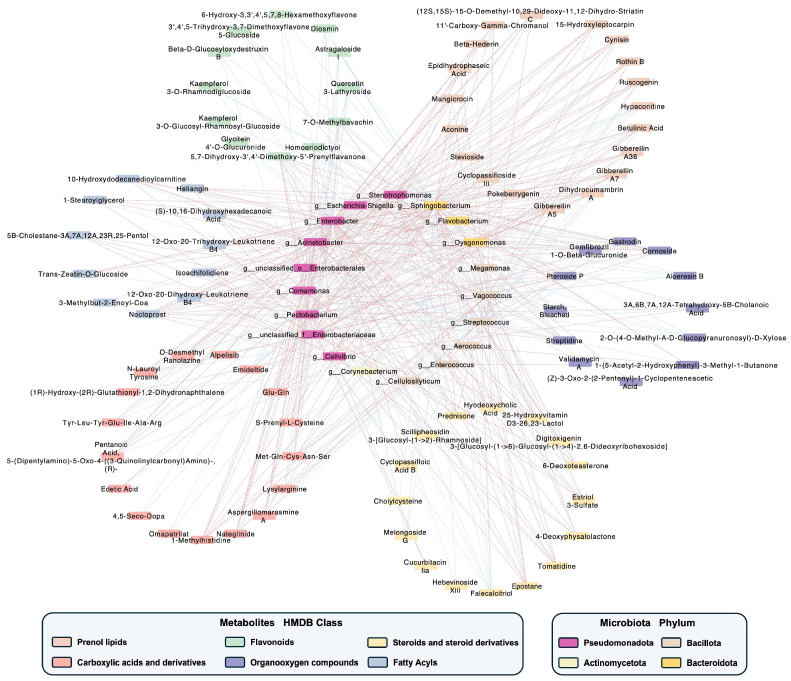
Correlation network analysis. The correlation between microbial abundance at the genus level and 89 differential metabolites across six major HMDB classes (prenol lipids, carboxylic acids and derivatives, steroids and steroid derivatives, flavonoids, organooxygen compounds, and fatty acyls) was performed. Edges indicate statistically significant Spearman correlations (*p* < 0.05, |r| > 0.7). Red edges represent positive correlations, while blue edges indicate negative correlations. *n* = 7.

To investigate the associations between microorganisms and metabolites in Typica Kopi Luwak beans following the drying process, a correlation analysis was conducted between major metabolite classes and microbial genera at the genus level using Spearman’s correlation coefficient (Figure 7).

Genera affiliated with the phylum Pseudomonadota, including *Stenotrophomonas*, *Enterobacter*, *Acinetobacter*, *Comamonas*, *Pectobacterium*, and *Cellvibrio*, exhibited strong positive correlations with most metabolites belonging to fatty acyls, carboxylic acids and derivatives, steroids and steroid derivatives, and prenol lipids.

*Sphingobacterium* and *Flavobacterium* were significantly positively correlated with prenol lipids and fatty acyls but negatively correlated with flavonoids. Meanwhile, *Dysgonomonas* showed broad positive associations with multiple metabolite classes, including prenol lipids (e.g., betulinic acid, dihydrocumambrin A, and cynisin), flavonoids (e.g., 7-O-methylbavachin), fatty acyls (e.g., 10-hydroxydodecanedioylcarnitine), and organooxygen compounds (e.g., pteroside P and cornoside).

*Streptococcus* displayed predominantly negative correlations with most metabolites across six major compound classes, whereas *Vagococcus* showed significant positive correlations with these metabolites, including prenol lipids (e.g., epidihydrophaseic acid and aconine), flavonoids (e.g., homoeriodictyol), and steroids and steroid derivatives (e.g., tomatidine).

*Corynebacterium*, the only genus from Actinomycetota identified in the correlation network, showed a negative correlation exclusively with Diosmin within the flavonoid class. Overall, strong associations were observed between microbial communities and metabolite profiles in Kopi Luwak beans before and after drying.

## 4. Discussion

The drying stage is recognized as the critical factor influencing coffee quality. Natural sun-drying is a traditional and cost-effective processing method in which whole coffee cherries are exposed to ambient conditions until the moisture content is reduced to approximately 10~12% [15]. During this process, environmental factors such as temperature, relative humidity, airflow, and the exposed surface area of the beans interact and may influence drying efficiency as well as the sensory attributes of the final product [16]. Kulapichitr et al. reported that sun-drying is associated with enhanced carbohydrate catabolism compared with mechanical drying and with alterations in amino acid profiles, which may contribute to the formation of key aroma precursors and improved flavor quality [17]. In addition, recent work by Zhu et al. [18] suggested that both fungal and bacterial communities are associated with flavor development during traditional sun-drying in Baoshan, Yunnan, with genera such as *Agaricus*, *Micromonospora*, and *Gilliamella* linked to sucrose and phenylalanine metabolism. Collectively, these findings indicate that the drying stage represents a key postharvest processing step as well as a dynamic phase during which microbial and metabolic changes are associated with the development of coffee quality attributes. Therefore, a deeper understanding of microbial and metabolite dynamics during drying may provide valuable insights to improve and control coffee quality.

Kopi Luwak is characterized by its unique production process involving biological transformation within the civet gastrointestinal tract [19,20]. In recent years, isolating and identifying microorganisms and enzymes from the civet gut for in vitro fermentation has attracted considerable attention as a strategy to improve coffee sensory quality [21,22]. However, studies focusing on postharvest processing of Kopi Luwak remain limited. Considering practical production conditions, natural sun-drying represents a key stage of interest in this study. It is hypothesized that interactions between residual gut-derived microorganisms and environmental microbes may occur during this process and potentially contribute to microbial succession. In the present study, 16S rRNA gene sequencing was employed to compare the bacterial profiles of Typica Kopi Luwak beans before and after drying. As shown in Figure 1, Figure 2 and Figure 3, clear separations in microbial structure, composition, and predicted functional profiles were observed between undried and dried samples, suggesting dynamic shifts in the bacterial community during the postharvest drying stage.

Notably, microbial dynamics during sun-drying have been investigated in conventional coffee systems [23]. For example, Florac et al. [24] reported that during the sun-drying of coffee cherries, *Acetobacter*, *Gluconobacter*, *Candida*, *Starmerella*, and *Saccharomycopsis* were among the dominant genera, accompanied by increased levels of metabolites such as gluconic acid and sugar alcohols. Similarly, in sun-dried Arabica coffee from Baoshan, the relative abundances of genera including *Agaricus*, *Micromonospora*, and *Gilliamella* were reported to increase, and these shifts were associated with sucrose and phenylalanine metabolism, potentially contributing to flavor-related metabolite formation [18]. In the present study, ANCOM analysis, which is considered to have a lower false-positive rate, identified *Streptococcus* (decreased after drying) and *Sphingobacterium* (increased after drying) as key differential genera between undried and dried Typica Kopi Luwak beans (Table 1). As *Streptococcus* is commonly associated with gut microbiota, its higher relative abundance in undried samples may reflect residual microbial signatures originating from civet digestion. *Sphingobacterium* has been reported to possess lignocellulolytic enzyme activity that can facilitate the saccharification of plant materials [25], which may be associated with the degradation of pectin and lignin, as well as the accumulation of fructose and mannose metabolites.

In addition, both pairwise comparison and LEfSe analyses identified several other potential differential genera, including *Enterobacter*, *Comamonas*, *Acinetobacter*, and *Pseudomonas*, which showed increased relative abundance after drying (Figure 2D and Figure 3B). Members of the genus *Enterobacter*, belonging to the family Enterobacteriaceae, have been reported to degrade pectin, galactose, fructose, and starch in coffee beans, and are associated with the production of compounds such as 2-isopropyl-3-methoxypyrazine, which contributes to potato-like flavor notes [26,27]. *Comamonas*, a genus widely distributed in natural and engineered environments, is capable of utilizing a broad range of organic substrates, including amino acids, carboxylic acids, sterols, and aromatic compounds [28]. Both *Acinetobacter* and *Pseudomonas* have been reported to participate in caffeine degradation [29]; however, compared to conditions where caffeine serves as the sole carbon source, the degradation rate by *Acinetobacter gerneri* KAFS 47 is reduced in the presence of glucose [30]. Taken together, these observations suggest that shifts in the relative abundance of these genera during the drying process may be associated with corresponding changes in metabolite profiles in Kopi Luwak beans.

The postharvest drying process is also accompanied by changes in metabolite composition [31]. For example, Zhu et al. reported that sun-drying was associated with enhanced catabolism of sucrose and phenylalanine in coffee beans [18]. In the present study, an untargeted metabolomics approach was applied to preliminarily investigate the impact of drying on the metabolic profiles of post-excretion Kopi Luwak beans. The results revealed clear distinctions in metabolite profiles between undried and dried samples (Figure 4A,B). KEGG enrichment analysis of the differential metabolites indicated that these changes were mainly associated with pathways such as fructose and mannose metabolism, valine, leucine and isoleucine degradation, and phenylpropanoid biosynthesis (Figure 5C). These pathway-level alterations may be linked to the observed increases in metabolite classes, including carboxylic acids and derivatives, organooxygen compounds, and coumarins and derivatives (Figure 6, Table S2), which may be associated with variations in coffee quality. However, as sensory evaluation was not performed in this study, the relationship between these metabolite changes and coffee quality remains to be further elucidated.

We further classified the potential differential metabolites between undried and dried coffee beans using the HMDB. Notably, alterations in the chemical composition of Kopi Luwak beans following drying were observed, particularly an increase in lipid-related metabolites such as prenol lipids and fatty acyls (Figure 6A,B,F). Compared with manually collected Robusta coffee beans, civet-derived coffee beans have been reported to contain higher levels of methyl octanoate, methyl decanoate, and total lipids, which are widely recognized for their flavor-enhancing properties and dairy-like aromatic notes [32]. Previous studies have reported elevated levels of total mono-chlorogenic acids and total chlorogenic acids in sun-dried green coffee beans [18,33]. Although chlorogenic acids were not directly annotated among the differential metabolites in this study, the increased abundance of phenolic compounds in the Typica_dry group (Table S2) may suggest potential accumulation or transformation of phenolic constituents during drying. In contrast, flavonoids are generally thermolabile and prone to oxidative degradation when exposed to heat and oxygen during drying [34], which may partially explain their reduced abundance in dried Kopi Luwak beans (Figure 6D; Table S2). No significant difference in caffeine content was observed between fresh and dried Kopi Luwak beans in this study, which may be related to the high chemical stability and resistance to degradation of caffeine [17,35,36]. These results suggest alterations in the metabolite profiles of Kopi Luwak beans before and after drying. However, given that untargeted metabolite annotation based solely on database matching does not constitute confirmed identification, future studies using authentic standards and targeted validation are required to verify metabolite identities.

As one of the most expensive coffee varieties globally, Kopi Luwak is limited by the activity cycle of the civet cat. Recently, in response to criticism from animal rights groups, the practice of mechanizing the civet’s digestive system to ferment coffee has emerged [37]. Additionally, foodborne pathogens present in civet feces could potentially transfer to the coffee beans, compromising the safety of subsequent processing [19]. Consequently, increasing attention has been given to the development of in vitro methods simulating civet coffee fermentation through enzymatic treatment and fermentation of fresh coffee cherries, offering a promising alternative to traditional production methods. This study analyzes the differential changes in microorganisms and metabolites in civet coffee beans during the drying process, providing new insights for in vitro simulation of coffee fermentation.

Despite these promising findings, our study has several limitations. The relatively small sample size and limited geographic and sampling scope may restrict the generalizability of the findings. In addition, the microbiome analysis was limited to bacterial communities based on 16S rRNA gene sequencing, without profiling fungal communities that may also influence fermentation and metabolite formation during the drying process, which warrants further investigation.

## 5. Conclusions

In conclusion, this study characterizes the changes in microbial community structure and metabolic composition of Typica Kopi Luwak beans during the drying process. These changes include the enrichment of specific bacterial genera, alongside alterations in key metabolic pathways related to lipid, carbohydrate, amino acid, and secondary metabolite metabolism. Integrative multi-omics correlation analyses suggest potential linkages between microbial dynamics and metabolite changes. Our findings highlight the potential effects of the post-excretion drying process on Typica Kopi Luwak beans, offering a foundation for future studies to explore causal relationships and to develop controlled or in vitro fermentation strategies for modulating coffee quality.

## Figures and Tables

**Table 1:** Differentially abundant genera identified by ANCOM in QIIME 2.

Feature	W	Reject Null Hypothesis	Typica_dry vs. Typica_fresh
*Streptococcus*	116	TRUE	down
*Sphingobacterium*	108	TRUE	up

The W value indicates the magnitude of difference between groups in ANCOM analysis, with higher values representing greater differences. “Reject null hypothesis” denotes statistical significance (TRUE = significant). “Typica_dry vs. Typica_fresh” indicates changes in the Typica_dry group relative to the Typica_fresh group.

## Data Availability

The original contributions presented in this study are included in the article/Supplementary Materials. Further inquiries can be directed to the corresponding author. The raw data of 16S rRNA gene sequencing and untargeted metabolomics reported in this study have been deposited in the GSA and OMIX, China National Center for Bioinformation / Beijing Institute of Genomics, Chinese Academy of Sciences (BioProject: PRJCA061024).

## Supplementary Materials

The following supporting information can be downloaded at: https://www.mdpi.com/article/10.3390/foods15081334/s1, Table S1. Validation parameters for the PLS-DA model. Table S2. Detailed VIP scores of individual differential metabolites categorized by HMDB class. Figure S1. Permutation test of the PLS-DA model.

## Abbreviations

The following abbreviations are used in this manuscript:

PCR	Polymerase Chain Reaction
ASVs	Amplicon Sequence Variants
Pd	Phylogenetic Diversity
PCA	Principal Component Analysis
ANOSIM	Analysis of Similarities
LDA	Linear Discriminant Analysis
LEfSe	LDA Effect Size
ANCOM	Analysis of Composition of Microbiomes
LC–MS	Liquid Chromatography–Tandem Mass Spectrometry
PLS-DA	Partial Least Squares Discriminant Analysis
HMDB	Human Metabolome Database
VIP	Variable Importance in Projection
FC	Fold Change
KEGG	Kyoto Encyclopedia of Genes and Genomes
SEM	Standard Error of the Mean
