# Supplementary material for: Comparative Microbiota and Metabolite Profiles of Undried and Dried Typica Luwak (Civet) Coffee Beans
Source: Foods. 2026 Apr 11;15(8):1334. doi: 10.3390/foods15081334 (PMC13114948; doi:10.3390/foods15081334)
Supplement: Supplementary file 1 [file foods-15-01334-s001.zip › foods-4208548-supplementary.pdf]

Supporting Information

Table S1. Validation parameters for the PLS-DA model.

	R^2X(cum)	R^2Y(cum)	Q^2	Q^2(cum)
P1	0.424	0.516	0.503	0.503
P2	0.608	0.919	0.8	0.901

R^2X (cum) and R^2Y (cum) represent the cumulative explained variance of the X and Y matrices by the model, respectively. Q^2 indicates the predictive ability of the model, where $Q^2 > 0.5$ suggests good predictive performance. P1 and P2 denote the first and second principal components, respectively.

Table S2. Detailed VIP scores of individual differential metabolites categorized by HMDB class.

Class	Metabolite	VIP	p-value	Typica_dry vs Typica_fresh
Glycerophospholipids	Glycerophosphoserine	2.8924	1.52E-13	down
	Gpcho(14:0/20:0)	2.5359	7.36E-06	up
	Glycerophospho-N-Arachidonoyl Ethanolamine	2.1502	0.00097	up
Phenols	Adipostatin A	2.4096	2.39E-08	up
	2-Methoxy-4-Vinylphenol	2.2389	7.60E-08	up
	Coniferyl Alcohol	2.1454	4.55E-11	up
Isoflavonoids	Biochanin A 7-(6-Malonylglucoside)	2.5144	6.39E-10	down
	6'-Malonyltrifolirhizin	2.2974	3.23E-09	down
Coumarins and derivatives	6'-O-Formylmarmin	2.441	4.71E-08	up
	Epoxybergamottin	2.3456	0.0002265	up

Sphingolipids	N-Acetylsphingosine	2.6294	0.0001018	up
Stilbenes	Dihydrodiethylstilbestrol	2.1108	0.0001296	up
Peptidomimetics	Didemnin A	3.007	2.04E-10	down
Lignan glycosides	Enterolactone 3''-Glucuronide	2.2611	6.87E-05	up
Indoles and derivatives	Methyl 5-Hydroxyoxindole-3-Acetate	1.4303	0.009121	up
Furanoid lignans	Matairesinol	2.8885	3.21E-07	up
Dioxepanes	Sporol	2.1448	0.0002137	up
Benzofurans	Peperinic Acid	2.9292	1.30E-08	up
Tropones	Colchiceine	2.6055	3.04E-11	up

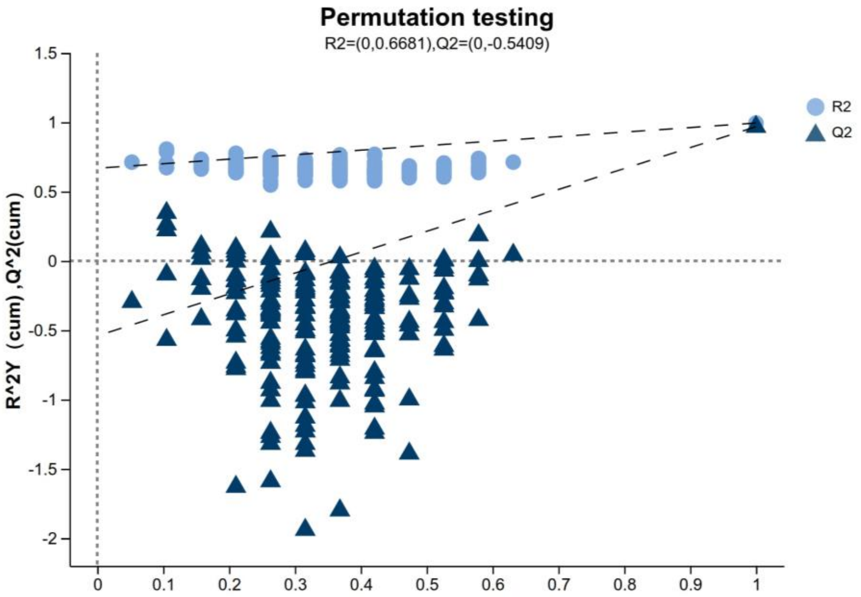

Figure S1. Permutation test of the PLS-DA model. The x-axis represents the correlation between the permuted and original class labels, and the y-axis represents the R^2 and Q^2 values obtained from the permutation test. The two dashed lines indicate the regression lines of R^2 and Q^2, respectively. A total of 200 permutations were performed. A Q^2 regression line intercept below 0.05 indicates that the model is robust and not overfitted.
